# 3D Visible‐Light Invisibility Cloak

**DOI:** 10.1002/advs.201800056

**Published:** 2018-04-14

**Authors:** Bin Zheng, Rongrong Zhu, Liqiao Jing, Yihao Yang, Lian Shen, Huaping Wang, Zuojia Wang, Xianmin Zhang, Xu Liu, Erping Li, Hongsheng Chen

**Affiliations:** ^1^ State Key Laboratory of Modern Optical Instrumentation Zhejiang University Hangzhou 310027 China; ^2^ The Electromagnetics Academy at Zhejiang University College of Information Science and Electronic Engineering Zhejiang University Hangzhou 310027 China; ^3^ Institute of Marine Electronics Engineering Zhejiang University Hangzhou 310058 China; ^4^ School of Information Science and Engineering Shandong University Jinan 250100 China

**Keywords:** 3D, invisibility cloaks, transformation optics, visible light

## Abstract

The concept of an invisibility cloak is a fixture of science fiction, fantasy, and the collective imagination. However, a real device that can hide an object from sight in visible light from absolutely any viewpoint would be extremely challenging to build. The main obstacle to creating such a cloak is the coupling of the electromagnetic components of light, which would necessitate the use of complex materials with specific permittivity and permeability tensors. Previous cloaking solutions have involved circumventing this obstacle by functioning either in static (or quasistatic) fields where these electromagnetic components are uncoupled or in diffusive light scattering media where complex materials are not required. In this paper, concealing a large‐scale spherical object from human sight from three orthogonal directions is reported. This result is achieved by developing a 3D homogeneous polyhedral transformation and a spatially invariant refractive index discretization that considerably reduce the coupling of the electromagnetic components of visible light. This approach allows for a major simplification in the design of 3D invisibility cloaks, which can now be created at a large scale using homogeneous and isotropic materials.

## 1 Introduction

Complex creatures, including humans, rely on their senses to obtain information about and react to their living environments. Visual illusions can affect the behavior of such creatures by deceiving their subjective judgment. Invisibility, one of the ultimate visual illusions, was almost inconceivable prior to the ingenious theory of transformation optics, which was first proposed in 2006.^1^, ^2^ Using transformation optics, a cloak could theoretically render an object invisible by guiding light around it as if nothing was there.

Despite its elegance, the proposed general cloaking theory^1^, ^2^, ^3^, ^4^, ^5^, ^6^, ^7^, ^8^ is tremendously difficult to implement; as a result, all previous implementations have involved special cases.^9^, ^10^, ^11^, ^12^, ^13^, ^14^, ^15^, ^16^, ^17^, ^18^ The first experimental validation of an invisibility cloak was a 2D microwave cloak^9^ for single‐frequency transverse electric (TE) waves. A nonmagnetic metamaterial cloak for transverse magnetic (TM) waves at microwave frequencies was subsequently proposed.^10^ Then, carpet cloaks were introduced; such cloaks are easier to implement because they are based on the principle of hiding an object under a reflective ground plane in a semispace.^5^ Carpet cloaks have been experimentally demonstrated in the microwave range^19^, ^20^, ^21^, ^22^, ^23^ and in the optical spectrum.^24^, ^25^, ^26^, ^27^, ^28^ However, carpet cloaks operate by manipulating reflected light to map an object to a “flat” surface instead of making an object “disappear” from transmitted light. To date, most experiments to verify that objects can be made to disappear from plain sight have been restricted to 2D cloaks because 3D cloaks are particularly challenging.

**Figure 1:**
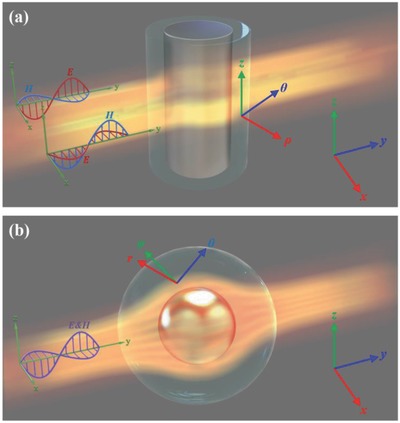
a) 2D cylindrical cloak for which waves with TE and TM polarizations are decoupled. b) 3D spherical cloak for which the polarizations of waves are coupled and are not easy to define.

Barriers to the implementation of 3D cloaks are associated with the complex anisotropic parameters required to simultaneously achieve both permittivity and permeability. In the case of a 2D cloak, such as the cylindrical cloak shown in **Figure** **1**a, waves propagate in a plane in which the TE and TM polarizations can be decoupled, which reduces complexity. For a TE wave with an electric field only in the *z*‐direction, this cloak requires only certain components of the permittivity and permeability tensors (μ_ρ_, μ_θ_, and ε_*z*_); conversely, for a TM wave with a magnetic field only in the *z*‐direction, the required components of these tensors are ε_ρ_, ε_θ_, and μ_*z*_. However, for a 3D cloak, such as the spherical cloak shown in Figure 1b, waves propagate in 3D space, where their polarizations are coupled and are not easy to define. Thus, a cloak must satisfy requirements for all parameters and is difficult to achieve. In certain special cases, such as situations involving DC and quasistatic spectra, the electric and magnetic fields can still be decoupled, and a 3D cloak can be attained by considering only permeability.^29^ In the regime of light diffusion, which leads to multiple light scattering, a 3D cloak can be achieved via the addition of diffusive shells.^18^ However, until now, a 3D cloak that functions for plain sight at optical frequencies had remained an unsolved problem.

## 2 Results and Discussion

Here, we achieve a 3D cloak for visible light by performing a 3D polyhedral transformation and using an approach involving a spatially invariant refractive index discretization. Our experimental demonstration shows that this cloak, which is made of isotropic materials, can hide macroscopic objects in fully polarized visible light. In contrast with a previous 2D cloak for the visible‐light spectrum,^16^ our cloak is effective for different viewing angles in 3D space.

**Figure 2:**
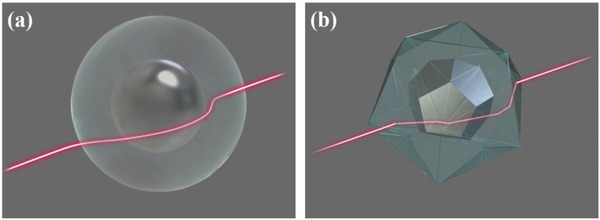
a) Spherical cloak that guides light smoothly around the hidden region. b) Polyhedral cloak that bends light at the boundaries of different segments to perfectly bypass the hidden region.

For a spherical cloak, light travels in an arc inside the cloak (**Figure** **2**a), and spatially inhomogeneous constitutive parameters are needed. Unlike traditional approaches, our polyhedral transformation method can be used to avoid this inhomogeneity. In fact, the use of different segments constructed with homogeneous constitutive parameters allows the trajectory of light to be controlled in a different way. Incident light bends at the interfaces of different segments in the cloak but can still perfectly bypass the hidden region (Figure 2b).

Although the polyhedral transformation eliminates the inhomogeneity of the material, anisotropic permittivity and permeability are still simultaneously required; satisfying this requirement is a major challenge with current metamaterial designs and high‐resolution 3D fabrication methods. Here, a spatially invariant refractive index discretization approach is used to replace the anisotropic indices with isotropic indices (see the Supporting Information). This approach is similar to the quasiconformal mapping method that was used to remove anisotropic constitutive parameters for the aforementioned carpet cloak design.^5^ One difference between these approaches is that the quasiconformal mapping in the carpet cloak design results in an inhomogeneous grid^5^ but the polyhedral transformation applied here generates a homogeneous grid for each segment. Moreover, unlike the carpet cloak, for which the reflection of light is considered and a line is transformed into another line, yielding moderate anisotropic parameters,^5^ the spherical cloak affects transmitted light and transforms a point into an inner sphere, resulting in highly anisotropic material parameters.^1^ Anisotropy is much greater for the grids in the spherical cloak than for the grids in the carpet cloak and cannot be directly ignored. Consequently, for the spherical cloak, replacing grids of highly anisotropic materials with grids of isotropic materials is no longer a valid option for omnidirectional incident light. However, our cloak can still guide light around a hidden object and make it return to its original path when the light is incident from certain 3D perpendicular directions. A detailed analysis is included in the Supporting Information.

**Figure 3:**
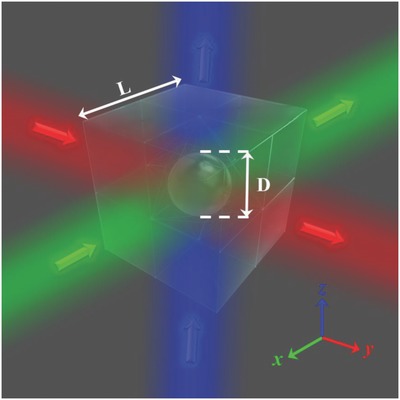
Schematic diagram of the simplified cubic cloak. This cloak is effective in three orthogonal directions in 3D space.

As a demonstration, a cloak with a cubic shape (**Figure** **3**) is fabricated. The cloak is constructed using two different types of optical glass. In particular, 12 tetrahedral pieces of glass (ZLaF78) with a permittivity of 3.61 are placed around the hidden region in the shape of a hexapod caltrop. The entire device is enclosed by eight heptahedral pieces of glass (ZBaF1) with a permittivity of 2.63, which matches the background permittivity. The regions between these glass segments are filled with water. The side length of the entire device is *L* = 100 mm, and the object to be hidden, a steel ball with a diameter of *D* = 38 mm, is placed in the device. The compression ratio is κ = 0.172 with *D*_1_ = 70.7 mm and *D*_2_ = 27.6 mm, respectively (see the Supporting Information). This cloak is operational for incoherent light illumination. Additional details regarding the cloak design and the calculated trajectory of the light propagating through the cloak are provided in the Supporting Information.

**Figure 4:**
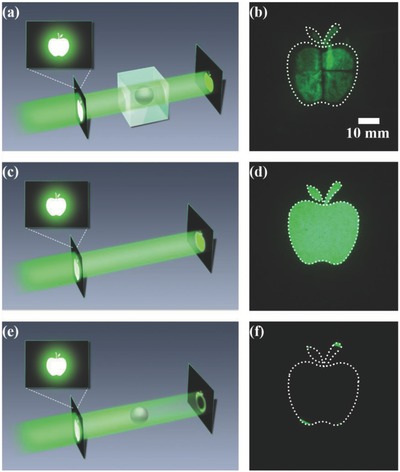
a) Experimental setup for measuring the cloaking effect. b) Captured image pattern for light passing through the steel ball covered with the designed cloaking device. The dotted line represents the outline of the mask's image pattern. c) Reference case with only the background and d) the corresponding captured image pattern. e) Reference case with only the steel ball and f) the corresponding captured image pattern.

In the experimental setup (**Figure** **4**a), the cubic cloak is vertically illuminated by a laser beam with a wavelength of 532 nm. The transmission pattern of an apple is placed between the laser beam and the device to serve as scenery to verify the cloaking effect. The light that is transmitted through the cubic cloak is projected onto a black screen, and the light pattern on the black screen is captured by a digital camera behind the screen.

Figure 4b shows the performance of this cubic cloak. When the steel ball is covered by the cubic cloak, the image pattern is recovered, and the steel ball is invisible to the observer. The cases with only the background (Figure 4c) and only the steel ball (Figure 4e) have also been measured for reference; the corresponding results are shown in Figure 4d,f, respectively. In the former case, the laser beam is transmitted through the area of interest and directly onto the screen, and the captured image pattern is well maintained. When the steel ball is placed between the mask and the screen without the cloaking device, most of the light is blocked, the image pattern is no longer maintained, and the steel ball is visible. In contrast, the designed cubic cloak clearly performs well. The recovered patterns are slightly distorted due to deviations in fabrication and edge effects but can be improved with the application of more accurate fabrication technology.

**Figure 5:**
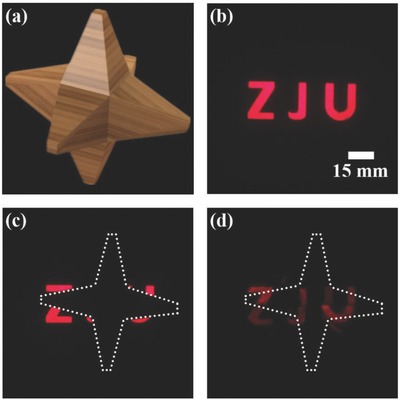
The cloaking effect of the designed cloaking device at different frequencies. a) The object to be hidden with a shape of hexapod caltrop. b) The captured image of the reference case with light transmitted directly from a transmission pattern of word “ZJU”. (c) The captured image of the reference case with light transmitted through the object. (d) The captured image pattern for light passing through the object covered with the designed cloaking device. The dotted line represents the outline of the hidden object.

In addition, **Figure** **5** shows the performance of this cubic cloak working at the wavelength of 650 nm. The transmission pattern is a word “ZJU” and the hidden object is in a shape of hexapod caltrop (Figure 5a). The captured images are shown in Figure 5b–d, respectively, which indicate the broadband cloaking performance for the designed cubic cloak.

It should be noted that in our demonstration, instead of using anisotropic materials, which are always highly dispersive, we use isotropic materials with very little dispersion; such materials can therefore be effective for the entire visible spectrum. Furthermore, since the cubic cloak is symmetric in three orthogonal directions in 3D space, this device works equally well in the three directions normal to the surfaces of the cubic cloak.

## 3 Conclusions

In conclusion, we propose a 3D cloaking device that can hide a macroscopic object from plain sight. Compared with 2D devices, our device offers an additional degree of freedom with respect to observation angles. Our work provides a new solution for hiding an object in 3D natural illumination for the entire spectrum of human eye sensitivity and will have practical applications in surveillance technology and for security‐ and defense‐related purposes.

## Conflict of Interest

The authors declare no conflict of interest.

## Supporting information

Supplementary
