# Supplementary material for: 3D Visible‐Light Invisibility Cloak
Source: Adv Sci (Weinh). 2018 Apr 14;5(6):1800056. doi: 10.1002/advs.201800056 (PMC6010732; doi:10.1002/advs.201800056)
Supplement: Supplementary file 1 — Supplementary [file ADVS-5-1800056-s001.pdf]

Copyright WILEY-VCH Verlag GmbH & Co. KGaA, 69469 Weinheim, Germany, 2018.

ADVANCED SCIENCE
Open Access

Supporting Information

for *Adv. Sci.,* DOI: 10.1002/advs.201800056

3D Visible-Light Invisibility Cloak

Bin Zheng, Rongrong Zhu, Liqiao Jing, Yihao Yang, Lian Shen, Huaping Wang, Zuojia Wang, Xianmin Zhang, Xu Liu, Erping Li, and Hongsheng Chen**

Supporting Information

Title: Three-dimensional visible-light invisibility cloak

Bin Zheng, Rongrong Zhu, Liqiao Jing, Yihao Yang, Lian Shen, Huaping Wang, Zuojia Wang, Xianmin Zhang, Xu Liu, Erping Li and Hongsheng Chen**

1. Polyhedral transformation for an ideal octahedral invisibility cloak

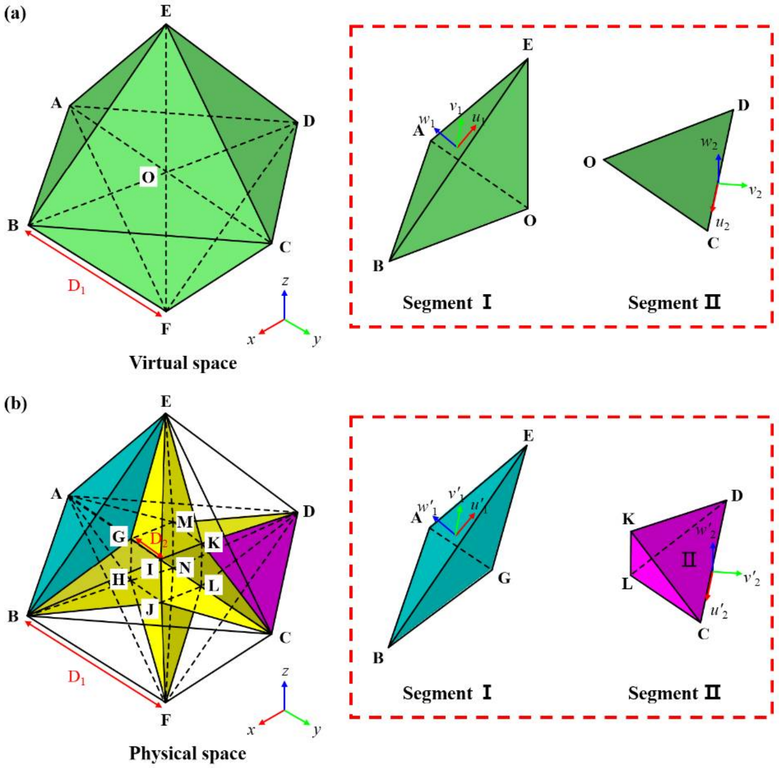

Figure S1. Schematic of a polyhedral transformation to design the cloak with (a) virtual space and (b) physical space. Each segment is transformed along its local coordinate axes. The segment of region I is transformed from a tetrahedron to another tetrahedron, while the segment of region II is transformed from a triangle to a tetrahedron. The hidden region is a hexapod caltrop shaped region which consists of one hexahedron and six pyramids. The side length of the outer octahedron is D_1 and the side length of the inner hexahedron is D_2.

In order to better illustrate our octahedral cloak design, we begin with a virtual space in the shape of an octahedron in free space, as shown in **Figure S1**a. The space is divided into 8 tetrahedral segments OABE, OBCE, OCDE, ODAE, OABF, OBCF, OCDF and ODAF (region I). There are also 12 triangular interfaces between every two adjacent tetrahedral segments, namely OAB, OBC, OCD, ODA, OAE, OBE, OCE, ODE, OAF, OBF, OCF and ODF (region II). The transformation is applied to each segment along its corresponding local coordinate axes, with compression or extension, leading to the physical space shown in Figure S1b. The segments of region I are transformed into the tetrahedral segments GABE, IBCE, KCDE, MDAE, HABF, JBCF, LCDF and NDAF, while the segments of region II are transformed into the tetrahedral segments ABGH, BCIJ, CDKL, DAMN, AEGM, BEGI,

CEIK, DEKM, AFHN, BFHK, CFJL and DFLN, respectively. The inset illustrates the transformation in detail, by showing the specific segments from each region. After the transformation, a hidden region in the shape of a hexapod caltrop is generated (yellow region in Figure S1b).

The transformation functions applied for different segments are:

$$\begin{aligned} &u_1' = u_1, v_1' = v_1, w_1' = \kappa w_1, \text{ for segments in region I} \\ &u_2' = u_2, v_2' = \kappa_a v_2, w_2' = \kappa_b w_2, \text{ for segments in region II} \end{aligned} \quad \text{(S1)}$$

where, κ, κ_a and κ_b are the compression or extension ratios with $\kappa = \left(\frac{\sqrt{6}}{6}D_1 - \frac{\sqrt{3}}{2}D_2\right) \Big/ \left(\frac{\sqrt{6}}{6}D_1\right)$, $\kappa_a = \left(\frac{1}{2}D_1 - \frac{\sqrt{2}}{2}D_2\right) \Big/ \left(\frac{1}{2}D_1\right)$ and $\kappa_b = \infty$, respectively.

The constitutive parameters are:

$$\begin{aligned} &\varepsilon_{1u}' = \mu_{1u}' = \frac{1}{\kappa}, \varepsilon_{1v}' = \mu_{1v}' = \frac{1}{\kappa}, \varepsilon_{1w}' = \mu_{1w}' = \kappa, \text{ for segments in region I} \\ &\varepsilon_{2u}' = \mu_{2u}' = \frac{1}{\kappa_a \kappa_b}, \varepsilon_{2v}' = \mu_{2v}' = \frac{\kappa_a}{\kappa_b}, \varepsilon_{2w}' = \mu_{2w}' = \frac{\kappa_b}{\kappa_a}, \text{ for segments in region II} \end{aligned} \quad \text{(S2)}$$

2. Spatial invariant refractive indices discretization

In Equation (S2), the parameters for segments in regions I and II are all diagonal tensors in their local coordinate systems. We denote g_u, g_v and g_w the principal values of the permittivity and permeability tensors, and we define the corresponding refractive indices to be $n_u = \sqrt{g_v g_w}$, $n_v = \sqrt{g_u g_w}$ and $n_w = \sqrt{g_u g_v}$. The refractive indices for each segment are:

$$\begin{aligned} &n_{1u}' = 1, n_{1v}' = 1, n_{1w}' = \frac{1}{\kappa}, \text{ for segments in region I} \\ &n_{2u}' = 1, n_{2v}' = \frac{1}{\kappa_a}, n_{1w}' = \frac{1}{\kappa_b}, \text{for segments in region II} \end{aligned} \quad \text{(S3)}$$

The anisotropy factor --- i.e., the maximum ratio of the refractive indices in different directions --- for each segment are calculated to be $\alpha_1 = \frac{1}{\kappa}$ (for segments in region I) and $\alpha_2 = \frac{\kappa_b}{\kappa_a}$ (for segments in region II), respectively. Since the grid obtained from the polyhedral transformation is homogeneous, the anisotropy factor in each segment is also homogeneous and it does not vary in space.

The anisotropy in the design of the octahedral cloak is eliminated with a spatial invariant refractive indices discretization approach. This approach is similar to the quasi-conformal

mapping method that has been used in the carpet cloak design[1] to minimize the average anisotropy factor in space. In that approach, the anisotropic refractive indices were replaced by isotropic ones, and the carpet cloak could be fabricated using dielectrics with an inhomogeneous refractive indices distribution. Although this approach leads to some lateral shifts,[2] the cloak could achieve an omnidirectional cloaking effect in a broad frequency band regardless of the light polarization.

In the designed octahedral cloak, since the anisotropy factors in the segments are not small enough, this approach influences the cloaking effect. Nevertheless, it is still valid for certain incident angles. We therefore replace the segments in the octahedral cloak with isotropic materials, as shown in **Figure S2**. The segments discretized with these spatial invariant refractive indices guide the light as designed in certain directions. For example, assume the incident light is propagating along the –z direction with $(\theta_0, \varphi_0) = (\pi, 0)$; then the isotropic segments with refractive indices

$$n_0 : n_I : n_{II} = 1 : \sqrt{\frac{2+\kappa^2}{3}} : 1+\kappa \tag{S4}$$

keep the same trajectories as the anisotropic ones.

However, from Equation (S4), we found that n_I should be smaller than unit, which is hard to realize in practice. This issue can be solved by introducing an additional type of segments (segments III), in the shape of heptahedrons, to act as background. As shown in Figure S2b, the three squared faces of each segment III are perpendicular to the x-axis, y-axis and z-axis, respectively. In this way, the ray trace of the light will still remain unchanged and the whole cloaking device can be effective in plain sight. With this addition, the refractive indices of segments I, II and III are:

$$n_I : n_{II} : n_{III} = 1 : \sqrt{\frac{3(1+\kappa)^2}{2+\kappa^2}} : \sqrt{\frac{3}{2+\kappa^2}} \tag{S5}$$

In the practical realization, the side length of the entire device is $L = 100$ mm, with $D_1 = 70.7$ mm and $D_2 = 27.6$ mm, respectively. The compression ratio κ is then calculated to be $\kappa = 0.172$ and the refractive indices $n_I : n_{II} : n_{III} = 1.333 : 1.9 : 1.621$. In addition, the volume of the total hidden region (a hexapod caltrop shaped region which consists of one hexahedron and six pyramids) is $V_{total} = \sqrt{2} D_1 D_2^2$, which is about 7.6% compare with the volume of the entire device.

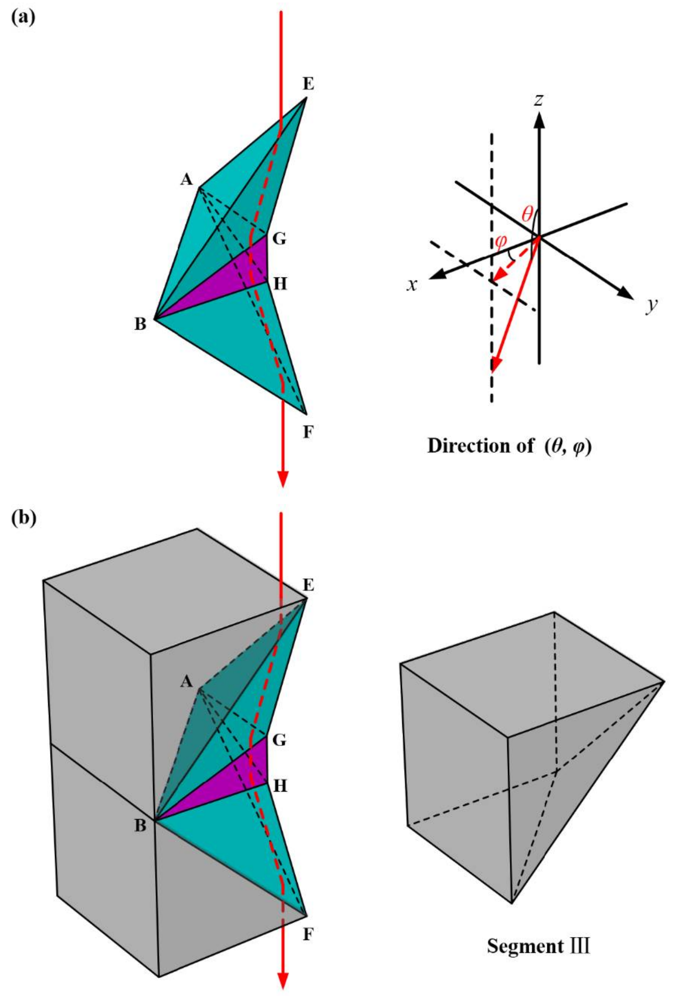

Figure S2. (a) Light propagating along the –z direction through specific segments. Each segment can be replaced by an isotropic material leaving the trajectory of light unchanged. (b) An additional type of segments (segments III) is applied to act as background. These segments do not affect the trajectory of light for light propagating along the main axes directions.

It should be noted that Figure S2a shows only part of the device, but due to its high symmetry, the approach will still be valid for light propagating along the –z direction from other parts of the device. In addition, for different directions $(\theta_0, \varphi_0) = (\pi/2, \pi)$ and $(\theta_0, \varphi_0) = (\pi/2, -\pi/2)$ --- i.e., along the –x and –y directions, respectively --- the device works equally well with the same refractive indices. Hence, the cloak simplified with this discretization approach can be effective for light that propagates along the main axes directions, where it is the most important in 3D space.

3. Calculation of the trajectory of light

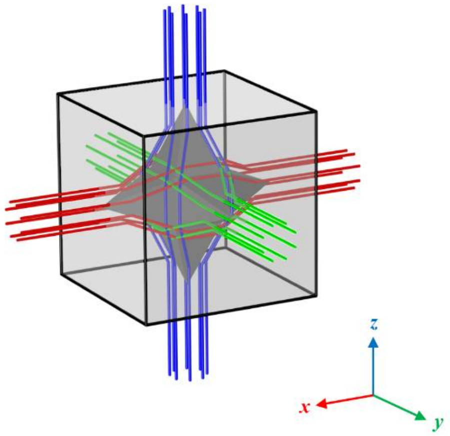

Figure S3. Calculated light ray trajectories in the simplified cloaking device.

The calculated trajectories of light in the simplified cloaking device are shown in **Figure S3**, where the red, green and blue lines represent light incident from different axes. For simplification, here the inner details of the cloaking device are omitted and only the outline of the simplified cloak is shown. Not only the light rays incident along the x-axis bypass the hidden object, but furthermore the trajectories of light in the simplified cloak are strictly the same as in the ideal one. Due to the structure symmetry, this simplified cloak is also effective for light incident along the y-axis and z-axis.

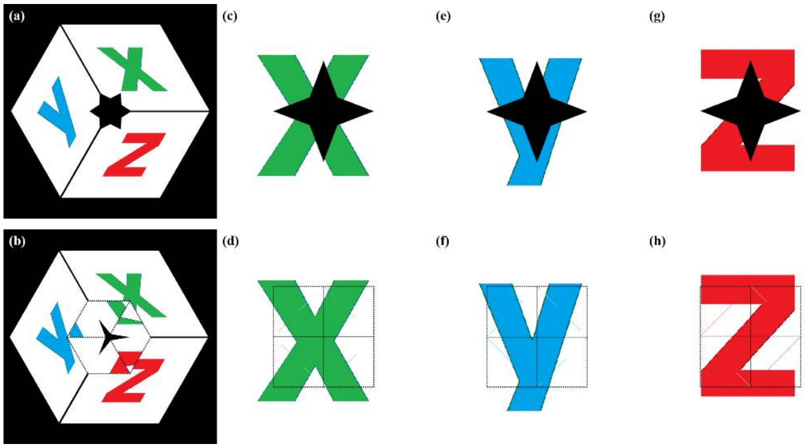

Figure S4. Virtual images obtained with numerical simulation. (a) The defined background image with an object placed in the middle. (b) The object is enclosed by the cloaking device. (c-h) Observing angles along the direction of the three orthogonal axes, without and with the cloaking device (top and bottom respectively). The dotted lines represent the outline of the cloaking device.

We performed a numerical simulation to validate the effectiveness of our cloaking device. As shown in **Figure S4**a, we define the background images along the direction of the x-axis, y-axis and z-axis, with the hidden object placed in the middle. All the segments of the cloak have been defined with shapes and refractive indices as discussed earlier. As shown in Figure S4b, the algorithm will display a virtual image by calculating the trajectory of light along a specific viewing angle. This algorithm does not take into account reflection, therefore the light blocked or totally reflected by the object will be shown in black.

Figures S4c to S4h show the virtual images calculated with this algorithm for viewing angles along the direction of the three orthogonal axes, without and with the cloaking device. We can see from these simulations that the background images are blocked by the object, but they are recovered when the cloaking device is in use, proving that our proposed cloaking device can be effective, for these specific angles, to guide the blocked light rays around the hidden object.

[1] J. Li, J. B. Pendry, *Phys. Rev. Lett.* **2008**, *101*, 203901.

[2] B. Zhang, T. Chan, B. I. Wu, *Phys. Rev. Lett.* **2010**, *104*, 233903.
